# Susceptibility and Antibody Response of the Laboratory Model Zebra Finch (*Taeniopygia guttata*) to West Nile Virus

**DOI:** 10.1371/journal.pone.0167876

**Published:** 2017-01-03

**Authors:** Erik K. Hofmeister, Melissa Lund, Valerie Shearn-Bochsler, Christopher N. Balakrishnan

**Affiliations:** 1 U.S. Geological Survey, National Wildlife Health Center, Madison, Wisconsin, United States of America; 2 East Carolina University, Greenville, North Carolina, United States of America; University of California Davis, UNITED STATES

## Abstract

Since the introduction of West Nile virus (WNV) into North America in 1999 a number of passerine bird species have been found to play a role in the amplification of the virus. Arbovirus surveillance, observational studies and experimental studies have implicated passerine birds (songbirds, e.g., crows, American robins, house sparrows, and house finches) as significant reservoirs of WNV in North America, yet we lack a tractable passerine animal model for controlled studies of the virus. The zebra finch (*Taeniopygia guttata*) serves as a model system across a diversity of fields, and here we develop the zebra finch a songbird model for WNV. Like many natural hosts of WNV, we found that zebra finches developed sufficient viremia to serve as a competent host, yet in general resisted mortality from infection. In the Australian zebra finch (AZF) *T*. *g*. *castanotis*, we detected WNV in the majority of sampled tissues by 4 days post injection (dpi). However, WNV was not detected in tissues of sacrificed birds at 14 dpi, shortly after the development of detectable anti-WNV antibodies in the majority of birds indicating successful viral clearance. We compared susceptibility between the two zebra finch subspecies AZF and Timor zebra finch (TZF) *T*. *g*. *guttata*. Compared to AZF, WNV RNA was detected in a larger proportion of challenged TZF and molecular detection of virus in the serum of TZF was significantly higher than in AZF. Given the observed moderate host competence and disease susceptibility, we suggest that zebra finches are appropriate as models for the study of WNV and although underutilized in this respect, may be ideal models for the study of the many diseases carried and transmitted by songbirds.

## Introduction

West Nile virus (WNV), a mosquito-vectored flavivirus of the Japanese encephalitis serogroup [1], was detected in North America following an epizootic in New York in 1999. Birds, such as hooded crows (*Corvus corone sardonicus*) and house sparrows (*Passer domesticus*) have been known since the mid—1950’s to amplify WNV in enzootic cycles involving mosquitoes as vectors [2, 3]. In North America a number of passerine bird species (songbirds, e.g., crows and relatives, American robins, house sparrows, and house finches) have been implicated as playing an important role in the maintenance of WNV in an enzootic cycle, yet, we lack a tractable experimental model of WNV in passerines.

A variety of birds have been experimentally challenged with WNV including wild-caught birds, wild birds maintained in captive breeding colonies, and poultry [4]. The use of captured wild birds is potentially compromised by stress hormone release associated with capture and confinement and concurrent infections, e.g. avian malaria that may confound the results of experimental challenge. To avoid this, captured wild birds are often acclimated for long periods and treated for possible concurrent diseases. Establishing a breeding colony with wild-caught birds, such as house sparrows, is difficult and not always successful. Additionally, the WNV susceptibility of the domesticated chicken and turkey has been examined [5–9] however, they are of low susceptibility to WNV making them less suitable as models for wild passerines infected with the virus.

One goal of an avian laboratory model of WNV might be to attempt to understand the susceptibility of a species which determines its relative role as a competent host capable of transmitting the virus to feeding mosquitoes. Alternatively, the goal might be to focus on disease resistance which protects the species from mortality and reduces the probability that it may serve as a competent host. With the goal of finding a passerine model of WNV that is relatively free of confounders and provides the ability, through breeding, to challenge birds of various age, sex, and genetic backgrounds, we examined the suitability of the zebra finch to fulfill that role. The zebra finch is a domesticated passerine that is easily maintained in captivity and is used frequently in biomedical research and, additionally, boasts a full suite of genomic resources [10]. Because of its role as a model system, the zebra finch was the second bird with a complete genome sequence and this genome remains one of the highest quality avian genomes [11].

A further objective of this study was to examine the susceptibility of adult male zebra finches of the two recognized subspecies, the Australian (AZF) and Timor zebra finches (TZF) (*T*. *guttata castanotis*, and *T*. *guttata guttata*, respectively) for West Nile virus. Our objective was to determine both the development of infection following challenge and the development of morbidity in infected birds. Timor zebra finches are a subspecies native to the Lesser Sunda Islands. The two subspecies are genetically distinctive [12, 13] with TZF showing a marked reduction in genetic diversity [12, 13], a feature that may compromise immune function.

## Materials & Methods

### Ethics Statement

All shipment of birds, daily husbandry, and study protocols were approved by the U.S. Geological Survey (USGS) National Wildlife Health Center (NWHC) Animal Care and Use Committee (Protocols EP030909A3 and EP120222).

### Zebra Finches and Animal Care

Zebra finches were either purchased from a commercial breeder or obtained from a breeding colony at the National Wildlife Health Center (NWHC) or the University of Illinois. All birds sourced from outside the Center colony were examined by the Center Veterinarian on receipt and held for 2 weeks in observation prior to WNV challenge. Any signs of clinical disease were brought to the attention of the Center Veterinarian. NWHC Colony birds were observed daily by the Center Veterinarian for clinical signs of disease and any mortalities were investigated by necropsy. Upon arrival at NWHC all birds were identified with a numbered leg band, and caged in the NWHC Biosafety Level 3 Animal Isolation Wing (AIW). Zebra finches were housed in 12 hr light: 12 hr darkness conditions in groups of 4–6 of the same sex in wire cages measuring approximately 60 cm (long) x 40 cm (wide) x 40 cm (height), and were provided a commercial finch seed mix (Vita Finch^®^, Vitakraft, Bowling Green, OH) and a captive small bird maintenance diet (Mazuri Small Bird Maintenance Diet^®^, Purina Mills LLC, St. Louis, MO) ad libitum. All birds were fed water ad libitum and supplied with a variety of wooden perches. Shipment, colony maintenance, study protocols were approved by the NWHC Institutional Animal Care and Use Committee. All tested birds were males, 6 months in age or older.

### WNV Dose-Response Study in Australian Zebra Finches

Three independent studies were conducted. In the first study, birds were randomly assigned to four treatment groups: procedural controls (n = 4), and three WNV challenge levels, 10^3^ (n = 14), 10^5^ (n = 13), and 10^7^ (n = 8) PFU (Table 1). One week prior to challenge, a 100 μL blood sample was obtained from the jugular vein of all birds for baseline WNV serology. Procedural controls were caged separately and on day 0 of the study were injected subcutaneously (s.c.) in the medial side of left thigh with 100 μL of BA1 media, as previously described [14]. West Nile virus challenge groups were inoculated s.c. with 100 μL of BA1 containing 10^7^,10^5^, and 10^3^ plaque forming units (PFU) of a low passage (< 3) 1999 American crow (*Corvus brachyrhynchos*) isolate of WNV (NWHC 16399–3) (Table 1). Three challenge levels of WNV inoculum span the range of virus inoculated by mosquito feeding (approximately 10^3^ to 10^6.6^ PFU of WNV) [15]. A bird was considered infected with WNV if the virus was cultured from its serum or oral swab sample at any sampling time point, or if the bird developed anti-WNV antibodies.

**Table 1:** Australian zebra finch susceptibility to infection is dependent on challenge level. Infection was determined by viral culture and by antibody development. Mortality and viral shedding are presented as the number of birds and the percent of the total number of birds infected.

Challenge Level	Challenged	Infected	Mortality	Viral Shedding	Mean Day of Peak Viremia (95% CI)
10^7^	8	8 (100)	3 (38%)	5 (63%)	2.3 (1.8–2.8)
10^5^	13	13 (100)	2 (15%)	2 (13%)	4.6 (3.6–5.5)
10^3^	14	9 (64)	0 (0%)	0 (0%)	4.9 (4.4–5.4)

Each of these inoculated groups was randomly divided into two sub-groups with the sub-groups sampled on an alternate day schedule on 1–8 days post-inoculation (dpi). All surviving birds were sampled on 10 and 14 dpi. An alternate day sampling schedule was used for the days 1–8 in order to minimize the effect of handling and sampling stress on the outcome of WNV challenge. Although sampling all alternate days reduces statistical power relative to daily sampling of each bird, such sampling is required under IACUC guidelines in order to yield sufficient blood volume for virus detection. On each sampling day, a 70 μL blood sample was obtained from the jugular vein and placed in BA1 media for a final serum dilution of 1:5, assuming a 50:50 ratio of red blood cells to serum in whole blood samples. Blood was allowed to clot in BA1 for 30 min at room temperature in CapiJect® tubes (Terumo Medical Corporation, Somerset, NJ) and then the sample was placed in wet ice until centrifugation at 2000 x g for 10 min. The resulting diluted serum samples were stored at -80 C until testing. Additionally, on each sampling date, an oropharyngeal sample was obtained using a sterile, cotton-tipped swab that was placed in 1 mL of BA1 medium, held on wet ice for approximately 30 min, and frozen at -80 C. Following WNV challenge, birds were observed twice daily for clinical signs of disease. Birds with difficulty perching, neurological signs of disease, or moribund birds were humanely euthanized. At 14 dpi all birds were euthanized by inhalation of CO_2_ gas.

### Development of Anti-WNV Antibodies and Tissue Distribution in Australian Zebra Finches

In a second study, 17 adult male AZF were inoculated with 10^5^ PFU WNV and two birds were injected with BA1 to serve as negative controls. In addition to the previous criteria for determining WNV infection, birds were tested for infection with WNV by culture of organ tissue. Three randomly selected birds were sacrificed at 2, 4, 7, and 14 dpi for necropsy and samples were obtained from skin, lung, liver, heart, spleen, kidney, and brain for virus culture. Two control birds were sacrificed and necropsied at 14 dpi. One additional bird euthanized at day 4 after showing clinical signs of disease. Serum was tested for the presence of anti-WNV antibodies at 2–5, 7, and 14 dpi. Surviving birds were euthanized at 14 dpi and were necropsied.

### Comparison of Australian and Timor Zebra Finches

In the third study, 16 adult male Timor zebra finches (TZF) were challenged with 10^5^ PFU of WNV and compared with a group of 23 adult male AZFs inoculated with the same challenge level. Six TZF were sacrificed at 2 and 4 dpi to compare tissue distribution of WNV following challenge. Additionally, to compare the WNV dose-response susceptibility of TZF, a group of 9 birds were challenged with 10^3^ PFU of WNV. Birds were divided into two groups for alternate day sampling, and were sampled 2–5, 10, 14 and 21dpi. In addition to blood and oropharyngeal samples, the birds were weighed on each sampling day on an electronic balance sensitive to 0.01 g. The relative susceptibility at each challenge level between subspecies and among the TZF was compared using mortality; viremia, as quantitated by RT-PCR; oral shedding of virus; body mass changes; and development of a specific antibody response to WNV.

### Virus detection by culture

Sera were cultured on confluent Vero cells (CCL-81, American Type Culture Collection, Manassas, VA), as described [16] at 1:10 dilution positive samples were titrated in a 2-fold dilution series until a countable endpoint was reached. Using this method, the limit of virus detection was 10^1.7^ PFU/mL. For calculation of group means for each sampling day, if a single member of the group was culture-positive, then culture negative birds were assigned 10^1.4^ PFU / mL for calculation of group means. If none were culture positive on a sampling day, then all were assigned 0 PFU/mL. Oral swabs were cultured at a 1:5 dilution in complete M199 on Vero cells grown on 6 well plates. A single overlay with neutral red was added [17] and, following inoculation, the cell cultures were observed for plaque development at 72–120 h and the number of plaques was recorded. Using this method the limit of virus detection was 10^1.0^ PFU/mL. Tissues were disrupted with 2.4 mm zirconia beads in a mini bead beater (BioSpec Products Inc., Bartlesville, OK) and blind passaged 3 times on Vero cells. Cultures demonstrating cytopathic effects were processed for viral detection by RT-PCR.

Immediately after euthanasia, the birds were necropsied and tissue samples were obtained from the skin covering the pectoral muscle, lung, heart, liver, spleen, kidney, and cerebrum and placed in 1 mL of BA1. In the third study, spleen samples were also placed in RNAlater (Qiagen Inc., Valencia, CA). Tissues in BA1 or RNAlater were frozen at -80°C for virus detection by culture and by RT-PCR.

### Virus Detection by RT-PCR

RNA was extracted from 140 uL of diluted serum in BA1 medium using the QIAamp Viral RNA Mini kit (Qiagen Inc.). RNA was extracted from brain and skin using the RNeasy Lipid Tissue, and RNeasy Fibrous Tissue Mini extraction kits, respectively (Qiagen Inc.), and from the remaining tissues using the RNeasy Mini extraction kit (Qiagen Inc.), as previously described [14]. A volume of 2.5 μL of extracted RNA was added to 22.5 μL of reaction master mix (QuantiTect Probe RT-PCR kit Qiagen Inc.) containing WNV-ENV forward and WNV-ENV reverse primers, as described [18]. Amplified product was detected using FAM–TAMRA probe (Integrated DNA Technologies, Coralville, IA) by an ABI Prism 7000 Real Time PCR Instrument (Applied Biosystems, Foster City, CA). In validation experiments using this protocol, samples with a cycle threshold (C*t*) of ≤ 34 were always positive for WNV when either culture of the same sample or another WNV E target primer set was used on extracted RNA, while those with C*t* scores ≥ 35 were always negative. Samples with C*t* between 34 and 35 were retested using conventional RT-PCR using the One Step RT-PCR Kit (Qiagen) and another set of WNV E primers, as described [16]. A negative control (dH_2_O) was included in each group of RNA extractions and RT-PCR reactions.

To conserve blood samples for serological assays, we used semi quantitative RT-PCR on RNA extracted from serum samples collected in the third challenge study. A mock zebra finch blood sample containing WNV was made by adding stock WNV isolate NWHC 16399–3, into negative control zebra finch serum, already at a 1:5 dilution in BA1. In parallel, a 140 uL aliquot of the mock sample was quantified by Vero cell plaque assay [18] and, from an additional 140 uL aliquot, the RNA was extracted as described, eluted in 60 uL of dH_2_O, and aliquots frozen at -80°C. To construct a standard curve for each RT-PCR run, a frozen aliquot of RNA was thawed, and a 10-fold dilution series containing 1.2 x 10^7^–1.2 x 10^−1^ genome equivalents (GE) of WNV/mL was made in dH_2_O, 2.5 μL of each dilution was added to reaction components (QuantiTect Probe RT-PCR Kit, Qiagen), and viral RNA was amplified, as described. These standards were assigned arbitrary units (AU) from 9–1. To test the matrix effect of zebra finch serum, this process was repeated with stock WNV diluted in BA1 media. Semi-quantitative RT-PCR reactions on the control series were performed in duplicate and were repeated once to assess within plate and between plate variability. For each RT-PCR assay a separate linear standard curve was determined from the estimated GE of control RNA dilutions as the independent variable and the instrument reported C*t* values as the dependent variable. The relative sensitivity of RT-PCR and viral culture for detection of WNV RNA was determined by performing both assays on sera obtained from American crows 4 dpi which were challenged with 10^5^ PFU of WNV isolate 16399–3. Details of RT-PCR validation are provided in the Supplementary Materia (S1 Fig).

### WNV Antibody Detection

All serum samples were heat inactivated at 56°C for 30 min and baseline and final serum samples were tested for anti-WNV antibodies by plaque reduction neutralization assay (PRNT) [17] on 12 well plates, using a 1999 American crow isolate of WNV, as described [19]. Samples exhibiting a neutralization of ≥ 90% were considered positive for antibodies to WNV (PRNT_90_). Additional, samples collected following WNV challenge were tested for specific anti-WNV antibodies by the anti-wild bird IgG enzyme immunoassay (WNV IgG EIA) [20], with minor modifications as previously described [21]. For the WNV IgG EIA, a low passage WNV isolate (16399–3) cultured in Vero cells was processed for positive antigen [22]. Similarly, negative control antigen was processed from unexposed Vero cells. Bound zebra finch antibodies were detected with horse radish peroxidase labeled polyclonal goat anti- wild bird IgG at a dilution of 1:2000 in wash buffer (Bethyl Laboratories, Cat. # A140-110P). To assess specific WNV antibody status, the ratio of the mean optical density (OD) of a sample recorded on wells coated with WNV antigen divided by the OD of the same sample recorded on wells coated with negative control antigen was calculated. Secondly, for samples in which the first ratio exceeded 2, the ratio of mean test serum OD to mean negative control serum OD (P/N), recorded on wells containing WNV antigen was calculated for each test sample. A P/N ratio of ≥ 2 was considered positive for anti-WNV IgG in a test sample.

In the third study the relative quantity of antibody to WNV at 10 and 21 dpi was determined using the WNV IgG EIA. For each EIA, a standard curve was created from a pool of AZF sera, previously determined to contain anti-WNV antibody, diluted to 1:50–1:800, and assigned EIA AU (5–1) of antibody reactivity. A single EIA plate was used for this comparison and a standard curve was created by determining the linear relationship between the mean OD value for each standard dilution and the units of specific antibody activity for each dilution.

### Statistical Analysis

For the first challenge study, daily mean, standard deviation (SD), and standard error (SE) PFU/ mL of log_10_ transformed viremia was calculated for each WNV challenge level [23] and plotted using the ggplot2 statistical package [24]. For peak viremia, the mean PFU/ml, SD, and SE of the highest detected viremia for each bird, by challenge level, was calculated using GraphPad Prism 5.00 Windows (GraphPad Software, La Jolla, CA). For categorical data, the proportion and 95% confidence interval (CI) were calculated and differences in proportions were compared with the Fisher’s exact test (GraphPad Prism 5.00 software). Unless indicated, all tests of proportions or means were two sided.

To assess the reproducibility of the standard curve for the linear relationship between C*t* and standard log_10_ GE, a linear model was used (lm function of R [23]) to determine coefficients for intercepts and slopes for the relationship between C*t* as a dependent variable and standard log_10_ GE, assay number, and the interaction term as independent variables. For each RT-PCR run, fitted values and SE for the mean response at each standard dilution were calculated and plotted using the gplots R package [25]. The coefficients of variation (CV) for replicated standards were determined (CV _intraassay_) and across all plates (CV _interassay_) using the high and low standard C*t* result. The potential matrix effect of zebra finch serum on the detection of WNV by RT-PCR was evaluated with the lm function of R with C*t* as the dependent variable and dilution of WNV standard, presence of serum, and their interaction as independent variables. For each RT-PCR run in the third challenge study, the linear relationship between the C*t* values, as dependent variable, and the standard log10 GE, as predictors, was determined (GraphPad Prism 5.00 software). Similarly, for the WNV IgG EIA, the linear relationship between the OD values and the EIA AU assigned to positive control serum dilutions was determined (GraphPad Prism 5.0 software).

## Results

### WNV Dose-Response Study in Australian Zebra Finches

#### Clinical disease in Australian zebra finches was challenge level dependent

**Fig 1:**
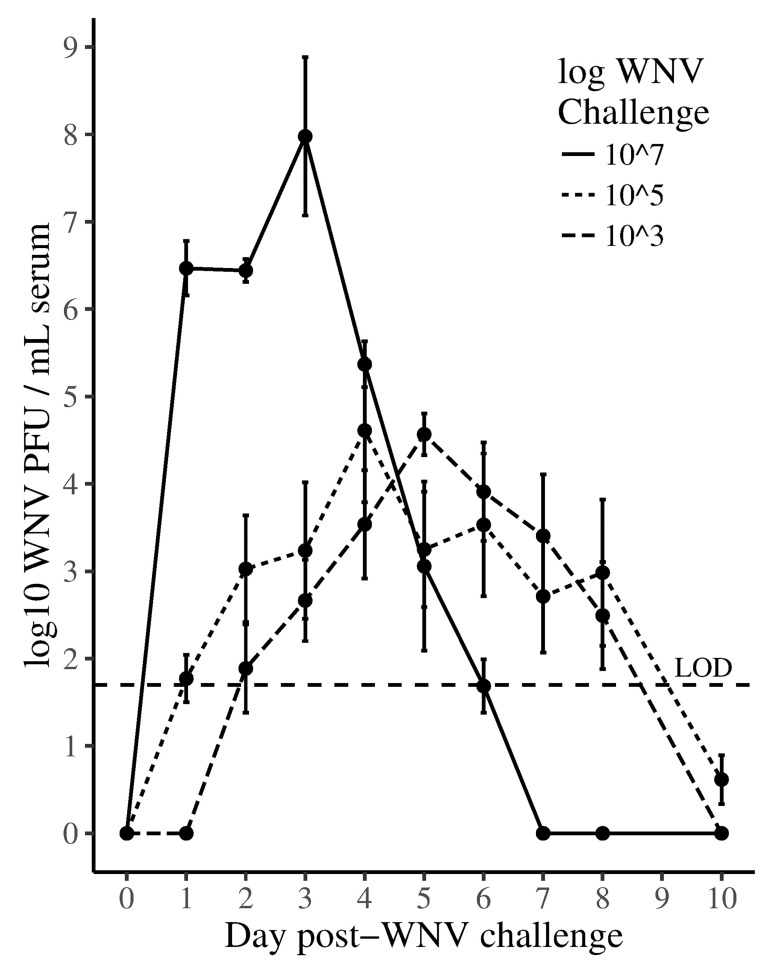
Australian zebra finches inoculated with 10^7^ plaque forming units (PFU) of WNV (solid line) had higher mean early log_10_ viremia and faster reduction of viremia than birds inoculated with 10^5^ (fine dashed line) or 10^3^ PFU (dashed line) of virus. Error bars represent standard error of the mean log_10_ PFU/mL serum. The horizontal dashed line indicates a limit of detection of 10^1.7^ PFU/mL.

In the first study, all AZF in the 10^7^ and 10^5^ PFU challenge groups became infected with WNV and developed viremia (Table 1, Fig 1). In contrast, WNV was cultured from significantly fewer birds in the 10^3^ WNV challenge group (7/14, 50%, CI 27–73%, Fisher’s exact test, p = 0.023 and p = 0.006, compared to the 10^7^ and 10^5^ PFU groups, respectively). Two additional birds in the 10^3^ PFU group developed anti-WNV antibodies (below) resulting in a total of infected birds that was significantly less than the 10^5^ challenge level (9/14, 64%, CI 39–84%, Fisher’s exact test, p = 0.04), but not the 10^7^ challenge group. All negative control birds (n = 4) were WNV negative by serology, culture, RT-PCR and none of them died.

Mortality was observed only in the 10^7^ and 10^5^ WNV challenge levels, (3/8 38%, CI 13–70%, and 2/13, 15%, CI 3–43%, respectively). Mortalities occurred at days 7, 8 and 10 in the 10^7^ PFU challenge and at day 10 in the 10^5^ PFU challenge (see S2 Fig). These low overall levels of mortality were not significantly different from the 10^3^ group where no birds died. Prior to 10 dpi, two birds in the 10^7^ challenge and a single bird died at 10^5^ challenge levels. Clinical signs of disease included respiratory difficulty, lethargy, inability to perch, and reduced appetite and fecal output.

WNV was detected only in swabs obtained from birds in the 10^7^ and 10^5^ challenge groups, (5/8, 63%, CI 30–87%, and 2/13,15%, CI 2–39%, respectively) (Table 1), a difference in proportions that was only marginally significant (Fisher’s Exact test p = 0.06), but WNV was shed orally in significantly more birds of the 10^7^ group as compared to the 10^3^ group (Fisher’s exact test, p = 0.009). WNV was shed orally in birds of the 10^7^ challenge group on multiple days from 3–7 dpi, whereas the virus was shed only on a single day in birds of the 10^5^ challenge group. Among birds in the 10^7^ challenge group, WNV oral shedding ranged from 101.70–10^3.24^ PFU per swab, while oral shedding in the 10^5^ challenge group was lower (10^1.70^–10^2.0^).

#### Peak viremia paralleled the WNV challenge level

Mean WNV viremia peaked at the highest level in the 10^7^ challenge group on 3 dpi (n = 4, mean = 10^7.97^ PFU/mL, SD 10^1.81^), followed by the 10^5^ group on 4 dpi (n = 7, mean = 10^4.61^ PFU/mL, SD 10^2.02^), and by the 10^3^ group on day 5 (n = 4, mean = 10^4.56^ PFU/mL, SD 10^0.48^), respectively (Fig 1). The mean peak day of viremia for individual birds of the 10^7^ challenge group (mean 2.3 d) occurred significantly earlier as compared to the other challenge groups (Table 1) (overall F = 10.03, p < 0.001, Tukey’s multiple comparisons of 10^7^ mean to 10^5^ and 10^3^, q = 5.58 and q = 5.24, respectively). Using the 10^4^–10^5^ PFU/ml criteria for avian WNV host competency [26], birds in the 10^7^ WNV challenge group might be infectious for feeding mosquitoes for 4 days (days 1–4) while birds in the other challenge groups might be infectious for only a single day. Viremia detected in birds of the 10^7^ WNV challenge group also fell to the threshold of detection (3 days) more rapidly than the 10^5^ or 10^3^ challenge groups (5 days).

#### Development of Anti-WNV Antibodies and Tissue Distribution in Australian Zebra Finches

Antibodies to WNV were not detected by PRNT, which detects neutralizing antibodies, in any baseline blood sample prior to WNV challenge. In the final serum sample collected at 14 dpi in the first study, the WNV IgG EIA, which detects any antibody to WNV, or PRNT_90_ detected antibodies to WNV in 8/8 (100%), 10/13 (77%), and 6/9 (66%) WNV infected birds in the 10^7^, 10^5^, and 10^3^ challenge groups, respectively. Additionally, in the 10^3^ challenge group, three WNV culture positive birds that were negative for anti-WNV antibody by WNV IgG EIA were positive by PRNT with the neutralization cut-off lowered to 80%.

In the second study, the virus was successfully cultured from serum or tissues in 11 of the 17 adult AZF challenged with 10^5^ PFU of WNV (65%, CI 41–83%). Additionally, anti-WNV antibodies were detected in an additional 4 birds resulting in total of 15 infected birds (88%, CI 66–97%). Mortality was not observed, nor was oral shedding of the virus detected in swabs obtained through 14 dpi. Anti-WNV antibodies were not detected by WNV IgG EIA on sera collected from 2–3 dpi. However, anti-WNV antibody was detected in 1/7 birds (14%) at 4 dpi 1/5 birds (20%) at 5 dpi, and in 6/10 birds (60%) at 7 dpi. All eight surviving birds were antibody positive at 14 dpi by both the WNV IgG EIA and PRNT with neutralizing antibody titers ≥1:80.

In the second study, WNV was detected by RT-PCR at 2 dpi in the skin of two of the three sacrificed zebra finches and in the spleen of one of these birds (Table 2). In contrast, by 4 dpi, multiple tissues removed from four sacrificed birds were WNV culture or RT-PCR positive. Kidney, liver, lung, and spleen were culture positive in all four birds sacrificed at 4 dpi. In addition at 4 dpi, WNV was cultured from brain and skin of one bird and detected by RT-PCR in both heart and skin samples from two birds that were culture negative in those tissues. At 7 dpi kidney, liver, and lung were uniformly culture or RT-PCR positive for WNV in all three sacrificed birds. The virus was also detected in a single bird by RT-PCR in brain and heart and by both detection methods in the spleen of another bird. By 14 dpi, WNV was not detected by either culture or RT-PCR in any tissue obtained from three sacrificed birds at this time point. None of the sacrificed birds for this analysis showed severe signs of clinical illness. As a comparison, we also examined the tissue distribution of WNV in four birds that showed severe clinical signs of infection and were sacrificed at 10 dpi. The severely affected birds were uniformly culture positive for WNV in kidney, liver, and lung and 75% were positive in heart, skin, and spleen tissues. One bird was culture positive and RT-PCR for WNV in brain tissue and the brain of two additional birds was RT-PCR positive for the virus. All negative control birds (n = 2) were negative by serology, culture, and RT-PCR.

**Table 2:** Post infection detection of WNV across tissue types. By 4 dpi WNV was detected by culture or RT-PCR from tissues obtained from all Australian zebra finches. WNV was not detected at all by day 14. N is the total number tested and each column indicated the number of positive within each tissue type.

DPI	N	Brain	Heart	Kidney	Liver	Lung	Skin	Spleen
2	3	0	0	0	0	0	2	1
4	4	1	1	4	4	4	3	4
7	3	1	1	3	3	3	1	0
14	3	0	0	0	0	0	0	0

### Comparison of Susceptibility of Australian and Timor Zebra Finches

Semi-quantitive RT-PCR revealed differences between TZF in AZF in their susceptibility to the virus. By day 5 dpi TZF at both the 10^5^ challenge (n = 5, mean = 4.66, SD 1.17, CI 3.20–6.12) and the 10^3^ level (n = 8, mean = 5.17, SD 1.67, CI 1.02–9.31), carried more virus than the AZF 10^5^ challenge (n = 11, mean = 2.40, SD, 1.49, CI 0.84–3.97) (Mann Whitney test, p = 0.052, p = 0.048, respectively). Accordingly, we also found that significantly more TZF challenged with 10^5^ WNV PFU (15/16, 94%, CI 70–99%, Table 3) developed circulating viral genomes that were detected by RT-PCR as compared to AZF inoculated with the same challenge level (12/23, 52%, CI 33–71%, Fisher’s exact test, p = 0.01, Table 3). Six AZF were negative for WNV by RT-PCR, however, developed antibodies. Thus, the total proportion of infected AZF (18/23, 78%, CI: 58–91%) is not different than the proportion of TZF determined to be infected by RT-PCR. Similar to the TZF challenged with 10^5^ WNV PFU, significantly more TZF challenged with 10^3^ WNV (8/9, 89%, CI 54—> 99%) had viral RNA that was detected by RT-PCR as compared to AZF at the 10^5^ challenge level (one sided—Fisher’s Exact p = 0.05).

**Table 3:** Patterns of infection, mortality, shedding and peak viremia in Australian versus Timor Zebra Finches. In comparisons of Australian and Timor zebra finches under the same challenge level (10^5^ PFU), Timor zebra finches show higher infection rates, mortality, and viral shedding, but these differences are not statistically significant.

Population	ChallengeLevel	N	Infected	Mortality	Viral Shedding	Mean Day of Peak Viremia (SD)
Australian	10^5^	23	18 (78%)	2 (11%)	0 (0%)	3.5 (1.1)
Timor	10^5^	16	15 (94%)	2 (22%)	1 (11%)	4.0 (0)
Timor	10^3^	9	9 (100%)	3 (33%)	2 (22%)	4.9 (0.7)

**Fig 2:**
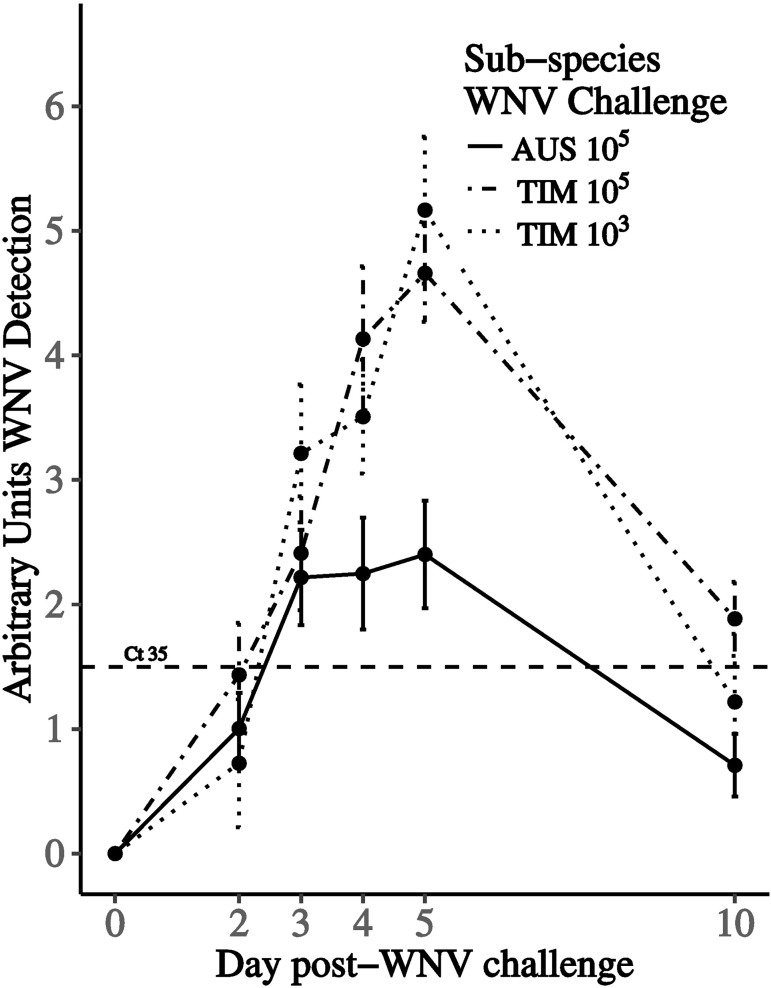
Relatively more WNV genome equivalents (GE) were detected by RT-PCR in serum samples from Timor zebra finches than in Australian zebra finches. Mean AU of detected GE and standard errors are plotted for Australian zebra finches challenged with 10^5^ plaque-forming units (PFU) of WNV (solid line), and for Timor zebra finches challenged with both 10^5^ (fine dashed line) and 10^3^ PFU of virus (dashed line). AU’s of 6–1 represent 1.2 x 10^4^–1.2 x 10^−1^ WNV GE/ mL.

Two of the nine TZF challenged at 10^3^ PFU that were RT-PCR positive for WNV at 10 dpi were euthanized due to the development of severe clinical signs of disease (C*t* = 24.8) or found dead (C*t* = 34) prior to 14 dpi. At the 10^5^ challenge level, mortality was observed in both AZF (2/18, 10%, CI 2–34%) and in two of the nine TZF (22%, 5–56%). Oral shedding of virus was not observed in AZF, but was detected in a single TZF at the 10^5^ challenge level and another two TZF at the 10^3^ challenge level. The mean day of WNV detection by RT-PCR, peaked slightly earlier in AZF than TZF (Table 3, Fig 2).

#### WNV Rapidly Disseminated to the Tissues of Challenged Timor Zebra Finches

At the 10^5^ PFU challenge level in birds sacrificed at 2 dpi, WNV RNA was detected by RT-PCR in two out of three birds in kidney and lung samples, and, additionally, in the spleen of one of these birds. Viral RNA was not detected in the third bird sacrificed at 2 dpi. In birds sacrificed 4 dpi following the same challenge, WNV was detected in the kidney and lung samples from all three birds, and in the spleen samples from two of the three birds. Tissues were not available at 14 dpi from TZF. However, three TZF were euthanized due to development of clinical signs of disease. The most severely affected bird was found on the cage tray liner at 10 dpi showing neurological signs of opisthotonus. Culture of brain, heart, kidney, liver, lung, spleen, and skin from this bird all revealed cytopathic effect (CPE) on Vero cells and WNV was detected by RT-PCR in lysed Vero cells. Histopathology of tissues obtained from the bird displaying signs of neurological disease showed multifocal mild to moderate signs of lymphoplasmacytic and heterophilic meningitis. Two TZF were euthanized at 14 days due to clinical signs of disease and in one bird CPE was uniformly detected in the same cultured tissues and WNV was verified by RT-PCR. In the second bird euthanized at 14 dpi, only brain tissue was available for culture, but CPE was also detected in Vero cells and WNV was verified by RT-PCR.

#### Antibody Response to WNV was Similar in Australian and Timor Zebra Finches

Despite the differences described above, antibody responses were similar between the two zebra finch subspecies. In the third study, anti-WNV antibodies were not detected by WNV IgG EIA in either AZF or TZF prior to 5 dpi. Anti-WNV antibody was detected in a single AZF (1/7, 14%, CI 4–58%) at 5 dpi and nearly all AZF had detectable antibodies to WNV by 10 dpi (17/18, 94%, CI 72—> 99%). Anti-WNV antibodies were first detected in TZF challenged with 10^5^ and 10^3^ PFU at 10 dpi (7/9 both groups, 78%, CI 44–96%), a difference that was not significant from AZF (Fisher’s exact p = 0.25). Using relative EIA AU for the WNV IgG EIA calculated from a standard curve derived from the same EIA run (data not shown), at 10 dpi, the mean EIA AU for AZF (n = 17, mean = 2.23, SD 1.55, CI 1.43–3.02) was greater than the mean for TZF (n = 10, 1.71, SD 0.80, CI 1.14–2.28). However, at 21dpi the mean AU for AZF (n = 9, mean = 2.81, SD = 1.19, CI 1.9–3.72) was slightly lower than the mean for TZF (n = 7, mean = 3.20, SD 0.63, CI 2.62–3.78). Neither of these differences was significant. Final serum PRNT titers at 21 dpi ranged from 1:20 to 1:320 in AZF (n = 10, geometric mean 6.92, mean titer = 177) and from 1:20 to 1:2560 in TZF (n = 7 geometric mean 7.46, mean titer = 121), a difference in mean titer that was not significant. At the 10^3^ PFU challenge level, anti-WNV antibodies were detected in the final serum sample (n = 9), however, the titers were not determined by PRNT_90_.

#### Loss of Body Mass Following Infection in Australian & Timor Zebra Finches

**Fig 3:**
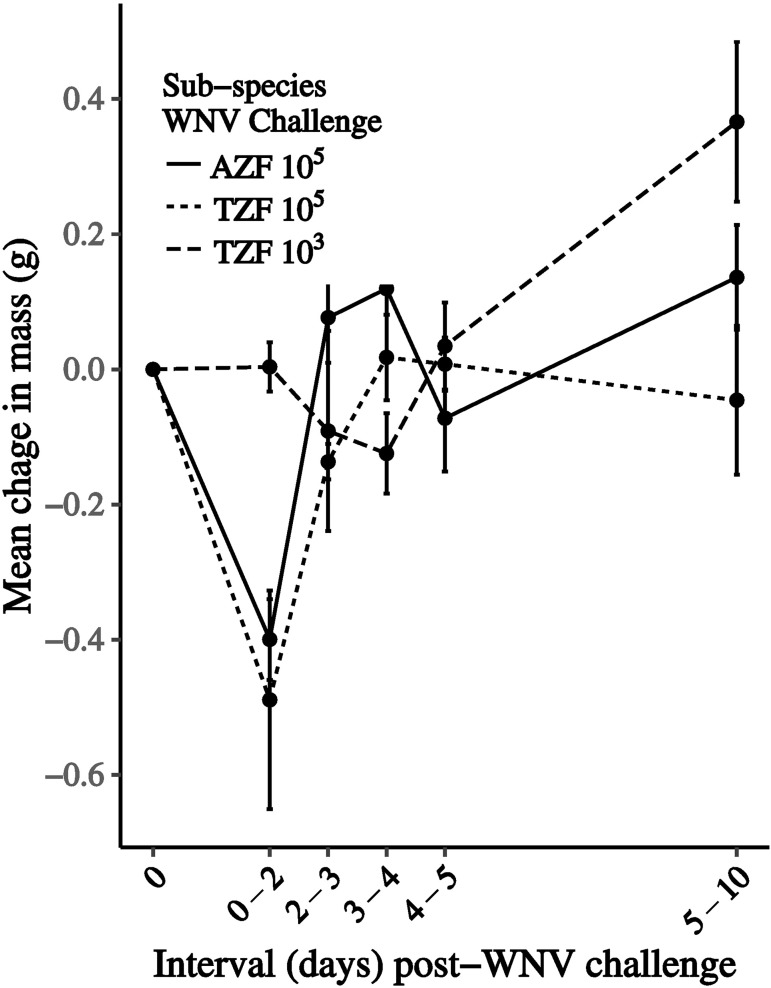
Change in mass following WNV challenge showed a dose response effect with birds challenged with 10^5^ plaque-forming units (PFU) of virus had earlier and greater loss of mass and a slower rebound to pre-challenge mass than birds challenged with 10^3^ PFU. Mean change in mass and standard error bars are plotted for daily intervals following challenge for Australian zebra finches challenged with 10^5^ plaque-forming units (PFU) of WNV (solid line), and for Timor zebra finches challenged with both 10^5^ (fine dashed line) and 10^3^ PFU of virus (dashed line).

Following WNV challenge a dose related loss of mass was detected in both Australian and Timor zebra finches (Fig 3). Both AZF and TZF challenged with 10^5^ PFU showed the largest drop in mass by 2 dpi and lost 3% and 5% of body mass, respectively. TZF challenged with 10^3^ PFU showed the biggest drop in mass at 4 dpi and lost 1% of mass on average. At the level of individual birds, the largest loss of mass was seen in a TZF at the 10^5^ PFU challenge. This individual lost 25% of its mass. The biggest mass loss in AZF was 7%. At 2 dpi, the loss in mass in birds challenged with 10^5^ PFU of virus significantly exceeded the loss in birds challenged with 10^3^ PFU of virus (F (2, 32) = 6.32, p = 0.005). By 10 dpi the mean mass had returned to within 5% of the starting mass (TZF 10^5^ PFU) or had exceeded the starting mass in the other groups.

## Discussion

Based on these studies, we find that domesticated zebra finches are clearly susceptible to infection by WNV, but are resistant to significant mortality. Furthermore, zebra finches develop sufficient viremia to serve as a competent host of WNV (>10 ^4^–10 ^5^ PFU/ mL of blood) [27–29]. Taken together, these features combined with their ease of care and breeding in the laboratory, make zebra finches an ideal model for study. In experimentally infected zebra finches dissemination of WNV was rapid, but birds that did not show clinical signs of disease were able to clear the virus from tissues by 14 dpi. Viral clearance from the tissues coincided with the development of an anti-WNV antibody response which was detected in the majority of birds by10 dpi.

Various experimental studies of WNV in captive wild birds have reported mixed results of a virus dose—clinical response relationship [4]. A clear dose response was reported in American robins [30]. However other experimental studies in cliff swallows (*Petrochelidon pyrrhonota*) challenged with 10^1–^10^3.5^ PFU of virus [31], and house finches and mourning doves (*Zenaida macroura*) challenged with 10^<0.3–^10^4.2^ PFU of virus [32] did not report a dose response in peak viremia. While viremia was higher in AZF challenged with 10^7^ PFU of WNV, this level of inoculation may be beyond the natural range that a songbird would face in nature (10^6.1^ PFU [15]). We did not observe a significant difference in viremia at the lower challenge levels. Similarly, in TZF we did not observe a significant difference in detection of WNV GE in birds challenged with 10^5^ or 10^3^ PFU of virus. However, in AZF we did detect a shift in the viremic period with the viremia detected in birds of the lower WNV challenge levels peaking later and lasting longer than birds with the 10^7^ PFU level. One might speculate that at the 10^7^ challenge level the higher antigenic mass of WNV may have led to either a more robust innate or acquired immune response. Changes in mass following WNV challenge might also reflect a dose response. A greater loss of mass in AZF and TZF was observed in birds challenged with 10^5^ PFU of WNV as compared to 10^3^ PFU. Loss of body mass has been reported in American robins experimentally challenged with ≤ 10^3^ PFU of WNV [30]. However, even at the 10^3^ PFU challenge we observed a loss in mass of up to 6% in an individual TZF that weighed approximately 9 gm at the start of the study. This loss occurred despite readily available food and might be more life threatening in free-ranging birds infected with WNV. Reduction in food and water intake and a reduction in activity characterize sickness behavior in birds and accompanies the acute phase response following infection [33]. These behavioral responses are thought to redirect energy away from foraging or growth and toward activities that promote recovery from infection and survival [34].

Unexpectedly, mortality was not observed in AZF challenged with 10^3^ PFU of WNV in our first study. In other WNV experimental studies in small wild caught passerines, using a comparable challenge by needle inoculation, the reported mortality ranged from as low as 10–15% in house sparrows [35–38] and 17% in song sparrows (*Melospiza melodia*) [39] to 63% in house finches [40]. In addition to inherent differences in species susceptibility to WNV, the confounding effect of stress may affect the survival from WNV challenge of wild-birds placed in captivity. Frequent handling and cage confinement of wild birds for blood collection has been reported to increase mortality with WNV in wild-caught birds acclimated to confinement [41]. Clinical signs of disease observed in zebra finches in our study are consistent with those reported in house sparrows [41] and have been reported to persist in house sparrows through 14 dpi [37]. We did not observe clinical signs of disease in any AZF after 10 dpi, but did observe signs of disease in three TZF after 10 dpi. While the number of birds tested was small, it appears that WNV is completely cleared from the tissues in AZF by 14 dpi. This is unlike the results reported for house sparrows in which WNV was isolated at 14 dpi from 3 of 8 challenged birds not showing clinical signs [41].

Our comparison of zebra finch subspecies suggests that TZF are more susceptible to WNV than are AZF. At the 10^5^ PFU challenge level a larger proportion of inoculated TZF had detectable virus in serum samples suggesting that they would be more competent as a host. A limitation to this finding is that RT-PCR does not distinguish between infectious and non-infectious virus particles and the result needs to be confirmed in subsequent studies using viral culture. Because a comparable WNV-specific antibody response developed in both AZF and TZF one might speculate that the difference in viral levels might be due to differences in the innate immune response. Gene expression studies in zebra finches indicate that as in mammals the RIG-I pathway, a key component of the innate immune response, is induced following WNV infection [42].

Many of the other comparisons of TZF and AZF are also suggestive of higher disease susceptibility, but fell short of statistical significance. While the sample size was small, we detected WNV by culture or RT-PCR in more TZF kidney and lung samples at 2 dpi than were detected in AZF at the same time after inoculation. Likewise a higher proportion of TZF (2/9, 22%) than AZF (2/18 11%) died following infection with 10^5^ PFU of WNV. Additionally, anti-WNV antibody was not detected in TZF prior to 10 dpi, whereas a small number of AZF had detectable antibody to WNV prior to 10 dpi and the proportion of AZF with detectable antibody at 10 dpi was larger, although not significantly, than for TZF. Timor and Australian zebra finches show significant allele frequency differences in the major histocompatibility class 1 gene [13] as well as in other genomic regions [12]. It is therefore possible that genetic differences mediate the observed differences in host competence, and the possible differences in disease susceptibility. AZF have also been domesticated for a longer time period and may be better adapted to captivity and therefore less stressed than their TZF counterparts. Adaptation to captivity may therefore also contribute to immune differences [43].

The advantage of using zebra finches as a model for WNV infection include the ability to conduct investigations into the molecular basis of host susceptibility to the virus and the ease with which the species can be maintained in a colony. By using a domesticated passerine species challenge studies of age related susceptibility may be conducted, as previously reported [44,45]. An obvious disadvantage of using zebra finches as a model is that in order to comply with animal care and use regulations only small blood samples (≤ 140 μL) may be obtained in non-terminal sampling. In order to obtain sufficient serum to be able to detect virus and perform serological assays we attempted, in study 3, to switch from viral culture to detection of virus by RT-PCR. However, as mentioned earlier, a disadvantage of using RT-PCR is that it may detect both infectious and non-infectious viral particles. Using blood samples from birds sampled in a previous study that provided sufficient serum for viral culture and RT-PCR, we found that the RT-PCR was less sensitive than culture. However, since the samples of the third study were all processed for RT-PCR and compared only within the study the results for that study are internally consistent. Another disadvantage in using AZF is there was some inherent variability in AZF challenged with the same WNV challenge. First, in viral culture of sera the variability in detection of WNV PFU was large. Second, in serological testing there also was variability among AZF challenged with 10^5^ PFU of WNV in the second and third studies. This variability might have had a genetic basis as domesticated zebra finches are generally maintained as outbred colonies with relatively high genetic diversity [40]. The colony that produced birds for these studies, however, was established from a limited number of breeding pairs. Anticipating this, as yet, unexplained variability one might increase the group sizes in future studies. If this variability has a genetic basis, this may actually provide yet another benefit to this system: the ability to genetically dissect the components of disease susceptibility.

Several questions specific to the use of zebra finches as an avian model of WNV remain that might be examined in future WNV challenge studies. First, was virus detected by RT-PCR at 10 dpi in the third study infectious? Culture of serum at 10 dpi could easily resolve this question. The sampling scheme used in these studies was based on the expectation that peak viremia would occur and be resolved by 8 dpi, therefore blood collected at 10 dpi was not routinely cultured. But culture of WNV from the tissues of a small number of TZF after 10 dpi and development of clinical disease suggests that some TZF may have patent viral infections beyond 10 dpi [35]. Second, relative susceptibility of AZF and TZF could be clarified by sacrifice of TZF at 7 and 14 dpi, as was done in AZF in the second study, and culture of harvested tissues. This would resolve whether TZF are slower to resolve viral infection as compared to AZF. This was not possible in the current study because an insufficient number of TZF were available. Last, while the number of TZF with clinical signs of disease after 10 dpi was small, our results suggest that WNV persists longer in TZF and the virus can still result in severe clinical disease after 10 dpi. This could be resolved with another comparative challenge of AZF and TZF.

Zebra finches have been used as models for studies of song learning, behavior, genetics, and development [10], but have rarely been reported as a model for the study of infectious disease. Previous reports have described using the zebra finch to study the effect of WNV on cytokine expression [44] and the age dependency of susceptibility of the species to WNV [45]. With these, our study is among the first to explore the use of domesticated zebra finches as a model for WNV research. Additionally, our study is the first to test the relative susceptibility of the two zebra finch subspecies to an infectious disease. Given the extensive laboratory resources for zebra finches including a high quality reference genome [11], genomic polymorphism data [46], cell lines [47,48], and developing transgenic techniques [49], zebra finches will be useful for ongoing studies of avian disease including West Nile virus.

## Supporting Information

S1 Table Linear regression coefficients for the model of cycle threshold scores (C*t*) as predicted by log_10_ WNV genome equivalents standards, the RT-PCR run number, and their interaction terms. (DOCX)

S1 Fig Linear regression of cycle threshold (C*t*), log_10_ GE standard, and RT-PCR run used to develop a standard curve for each amplification run. Plotted are the predicted values and standard errors. According to the model runs 2 had a significant intercept coefficients (p = 0.02). Intercept and slope estimated coefficients for the remaining runs were not significant. A separate linear regression was used for each RT-PCR run as a calibration curve.
(EPS)

S2 Fig Survival of Australian zebra finches following experimental inoculation with West Nile virus. Survival of birds inoculated with 107 plaque forming units (pfu) of virus was not significantly different from birds inoculated with 103 pfu of virus (Log-Rank Test Chi-sq 3.4, df = 1, P = 0.07). (EPS)
