# Supplementary material for: Susceptibility and Antibody Response of the Laboratory Model Zebra Finch (Taeniopygia guttata) to West Nile Virus
Source: PLoS One. 2017 Jan 3;12(1):e0167876. doi: 10.1371/journal.pone.0167876 (PMC5207765; doi:10.1371/journal.pone.0167876)
Supplement: S1 Table — (DOCX) [file pone.0167876.s001.docx]

**S1 Table.** Linear regression coefficients for the model of cycle threshold scores (C*t*) as predicted by log_10_ WNV genome equivalents standards, the RT-PCR run number, and their interaction terms.

| Coefficient | Estimate | Std. Error | Pr > t value |
| --- | --- | --- | --- |
| Intercept | 30.28 | 0.17 | < 0.0001 |
| log_10_GE | -3.43 | 0.07 | < 0.0001 |
| RT-PCR Run 2 | -0.52 | 0.24 | 0.05 |
| RT-PCR Run 3 | 0.24 | .24 | 0.33 |
| RT-PCR Run 4 | 0.34 | 0.24 | 0.17 |
| RT-PCR Run 5 | -0.59 | 0.24 | 0.02 |
| log_10_GE*Run 2 | 0.17 | 0.10 | 0.10 |
| log_10_GE*Run 3 | 0.16 | 0.10 | 0.13 |
| log_10_GE*Run 4 | -0.17 | 0.10 | 0.10 |
| log_10_GE*Run 5 | 0.09 | 0.10 | 0.38 |
